# ADVANCING HOME MODIFICATION SERVICE DELIVERY THROUGH SYNTHESIZING DATA AND EVIDENCE

**DOI:** 10.1093/geroni/igae098.0006

**Published:** 2024-12-31

**Authors:** Bernard A Steinman, Scott Trudeau, Maria Henke

**Affiliations:** Chair; Co-Chair; Discussant

## Abstract

Supportive housing options are a cornerstone to aging in place. Whereas most older people prefer to remain in their homes as they age, challenges associated with aging, including declines in physical health and functioning, often act as barriers to meeting this goal. Additionally, the home’s condition can affect a person’s ability to care for themselves and the ability of caregivers to provide quality care. Home modifications (HM) that increase safety and support daily activities play a key role in helping people stay in their homes and communities. Indeed, evidence-based interventions with HM components have been shown to reduce falls-related accidents, address a range of caregiving needs, and delay or prevent institutionalization–outcomes that otherwise come with high costs to individuals and society. In this symposium we have three aims: 1) to summarize previous research supporting the efficacy of programs with HM components in promoting aging in place via improved safety and access in the home; 2) to present three original studies that incorporated data from the National Health and Aging Trends Study (NHATS) to reveal HM use patterns (e.g., whether HMs are implemented proactively in advance of acquiring disability, or reactively in response to acquiring disability); and 3) to introduce a current HM service delivery model, describe its development, and discuss implications and next steps, including how current research findings can inform service delivery strategies in the future.

